# Traumatic arteriovenous fistula after ice axe injury 27 years ago: A case report

**DOI:** 10.1113/EP093968

**Published:** 2026-07-27

**Authors:** Kate N. Thomas, Emma M. W. Jones, Arunesh Majumder, Peter W. Abcarian, Peter J. McLeod

**Affiliations:** ^1^ Department of Surgery and Critical Care, Dunedin School of Medicine University of Otago Dunedin New Zealand; ^2^ Dunedin Hospital, Health New Zealand Te Whatu Ora – Southern Dunedin New Zealand

**Keywords:** case report, flow‐mediated dilation, shear stress, shunt, vascular remodelling

## Abstract

We report a case of a traumatic arteriovenous fistula (AVF) between the superficial femoral artery and femoral vein that went undetected for 27 years following an ice axe injury during a climbing accident in 1997. An AVF is an abnormal connection between an artery and a vein, where blood flows directly from the high‐flow arterial system to the low‐flow venous system, bypassing the capillaries. A 74‐year‐old male presented with a 2‐year history of extensive left leg swelling. He had visible posterior thigh and calf varicose veins and a small scar to the left medial upper thigh. He was otherwise fit and hiking regularly. He also had mild left ventricular and severe bi‐atrial dilation, with high output cardiac failure and cardiac remodelling. With ultrasound, a traumatic AVF between the superficial femoral artery and femoral vein was identified. We also assessed the arterial vasodilatory function in both femoral arteries for a within‐subject comparison of the chronic effects of high shear stress – the result of high flows through the AVF. This case study discusses the imaging and physiological test findings, along with further details of the climbing accident which were revealed by the patient. Traumatic AVFs are uncommon, and to our knowledge, this is the first documented case of an AVF resulting from an ice axe injury. Almost three decades later, the moment on the mountain left its mark; this case illustrates the complications associated with an untreated AVF.

## Abstract (highlights)

- **What is the main observation in this case?**
  An arteriovenous fistula resulting from an ice axe injury to the thigh during a climbing accident went undetected for ~27 years.
- **What insight does it reveal?**
  The case demonstrates the vascular remodelling and clinical complications of chronic, high‐flow shunting between the artery and the vein.

## 1 INTRODUCTION

We report a case of a traumatic arteriovenous fistula (AVF) between the superficial femoral artery (SFA) and femoral vein (FV) that resulted from an ice axe injury during a climbing accident in 1997 and went undetected for 27 years. An AVF is an abnormal connection between an artery and a vein, where blood flows directly from the high‐flow arterial system to the low‐flow venous system.

## 2 PARTICIPANT INFORMATION

A 74‐year‐old male (Mr G) who had regularly exercised at a moderate to high workload throughout his life, including hiking, cycling and running, was referred to the cardiology clinic with a new diagnosis of atrial fibrillation and a drop in exercise tolerance due to breathlessness and fatigue. He had noted intermittent left foot swelling which had become more persistent at the time of referral. His general practitioner trialled furosemide (diuretic) with little improvement in symptoms.

**FIGURE 1:**
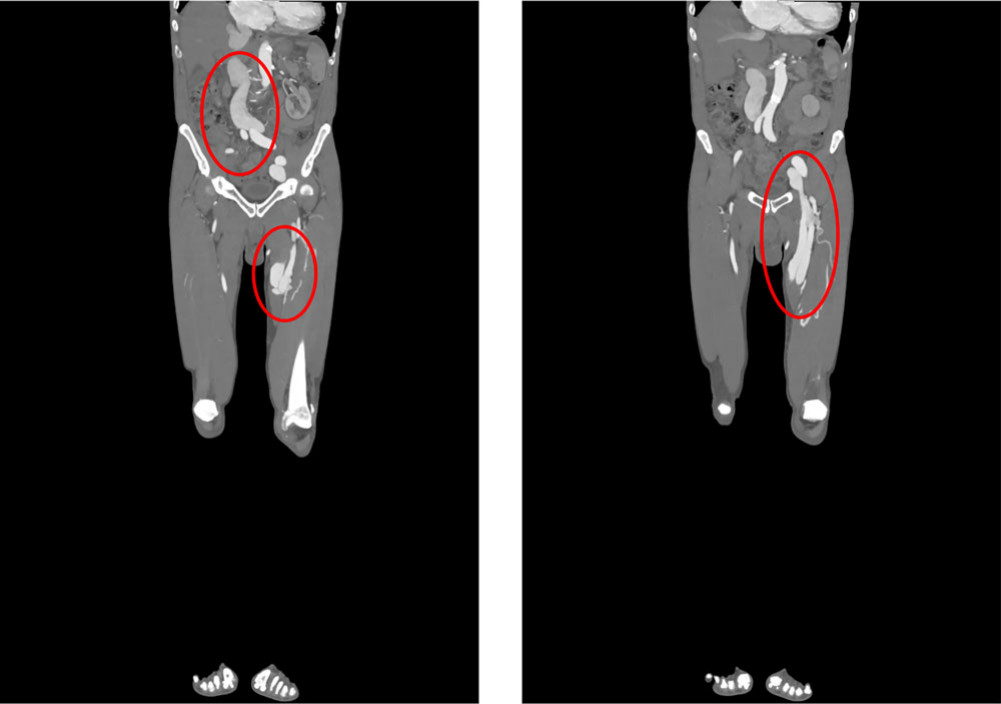
CT images demonstrating dilated inferior vena cava and left iliofemoral veins.

In the cardiology clinic, the peripheral oedema was noted to be mild and unilateral, jugular venous pressure was not elevated and there were no signs of pulmonary congestion. His echocardiogram demonstrated severe bi‐atrial dilation, mild to moderate left ventricular dilation, and mild right ventricular dilation with normal bi‐ventricular function. There was no haemodynamically significant cardiac valve disease. There were no clear signs of heart failure and therefore the furosemide was stopped. Subsequently the peripheral oedema increased significantly involving the entire left leg to the groin with the development of a hydrocele (scrotal fluid distension). Mr G did not experience any change in respiratory symptoms or pulmonary congestion to suggest this was due to decompensation of heart failure, and echocardiography revealed a preserved ejection fraction. As part of the work up for the hydrocele, Mr G underwent a CT of his abdomen and pelvis which demonstrated generalised dilatation of the inferior vena cava (IVC) and marked dilatation of the left iliofemoral veins without an obstructive cause (Figure 1). Mild ascites was also present, and the radiological impression was of congestion due to cardiac causes. A high cardiac output state was suspected on cardiac MRI (left ventricle: 9.1 L/min), and alongside the findings of four chamber cardiac enlargement, unilateral lower extremity oedema and markedly enlarged IVC and left iliofemoral veins, a high cardiac output state due to a lower extremity arteriovenous fistula was suspected. He was therefore referred to Vascular Surgery for further diagnostic imaging and ultimately for definitive treatment.

**FIGURE 2:**
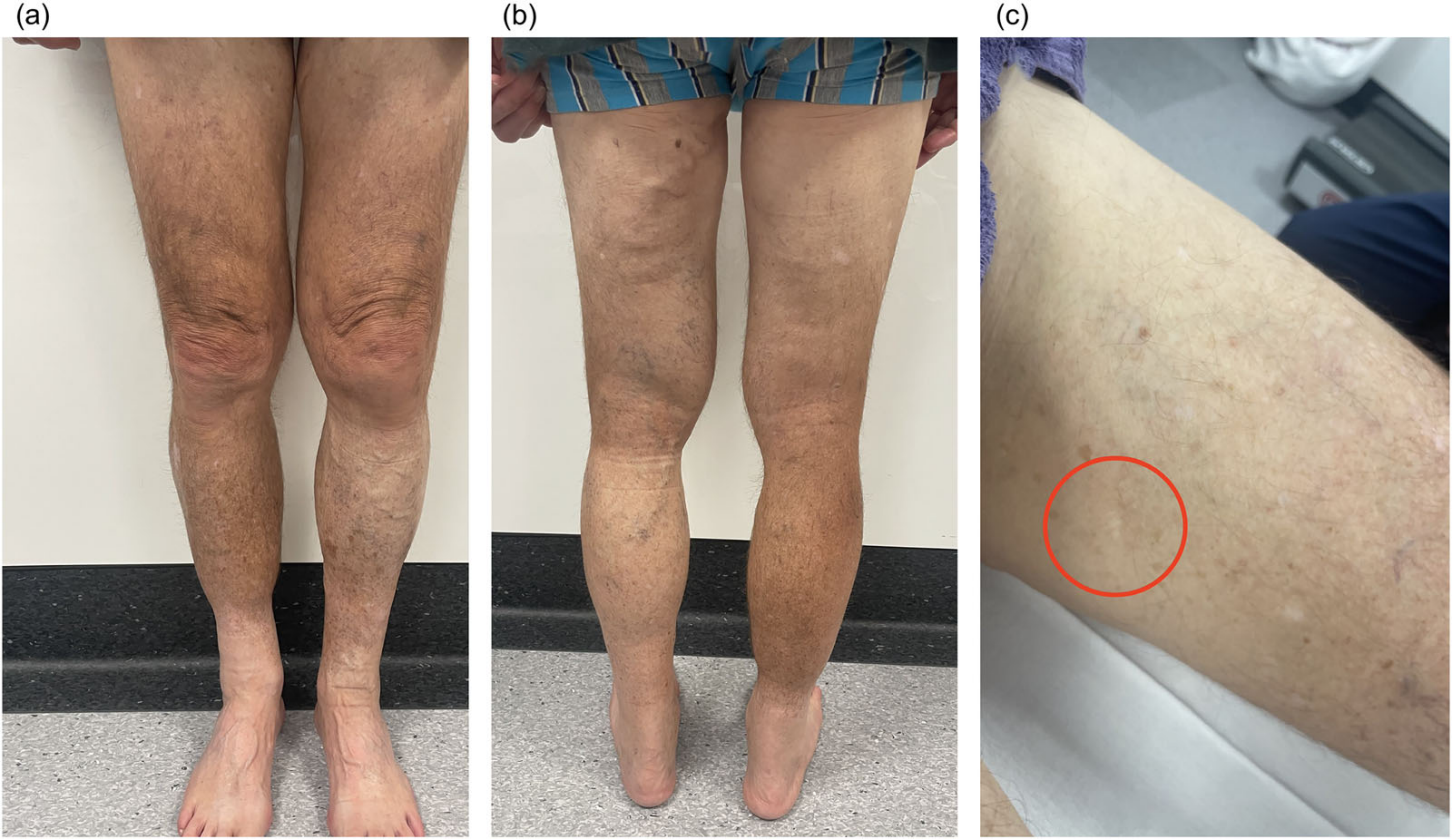
Photos of Mr G at presentation demonstrating left leg swelling (a, b), posterior left thigh varices (b) and left thigh scar (c).

Mr G was then referred for a venous ultrasound assessment to identify any venous pathology associated with the generalised dilatation. Measurements of his legs were unremarkable, with the left measuring 2 cm greater in circumference at the level of the distal thigh, and 1 cm greater in the calf, with no difference in the upper thigh, calf or ankle (Figure 2a). Visible posterior thigh varices were seen on the left (Figure 2b) with mild skin changes in the calf, and a small scar on the medial left upper thigh (Figure 2c).

During the ultrasound examination, when questioned about any previous injury to the left leg, Mr G reported a climbing accident 27 years ago resulting in ice axe punctures to the face and left thigh. He had felt a thrill at the site of the thigh injury since the accident. He had diminished distal pulses but no overt signs or symptoms of chronic ischaemia.

**FIGURE 3:**
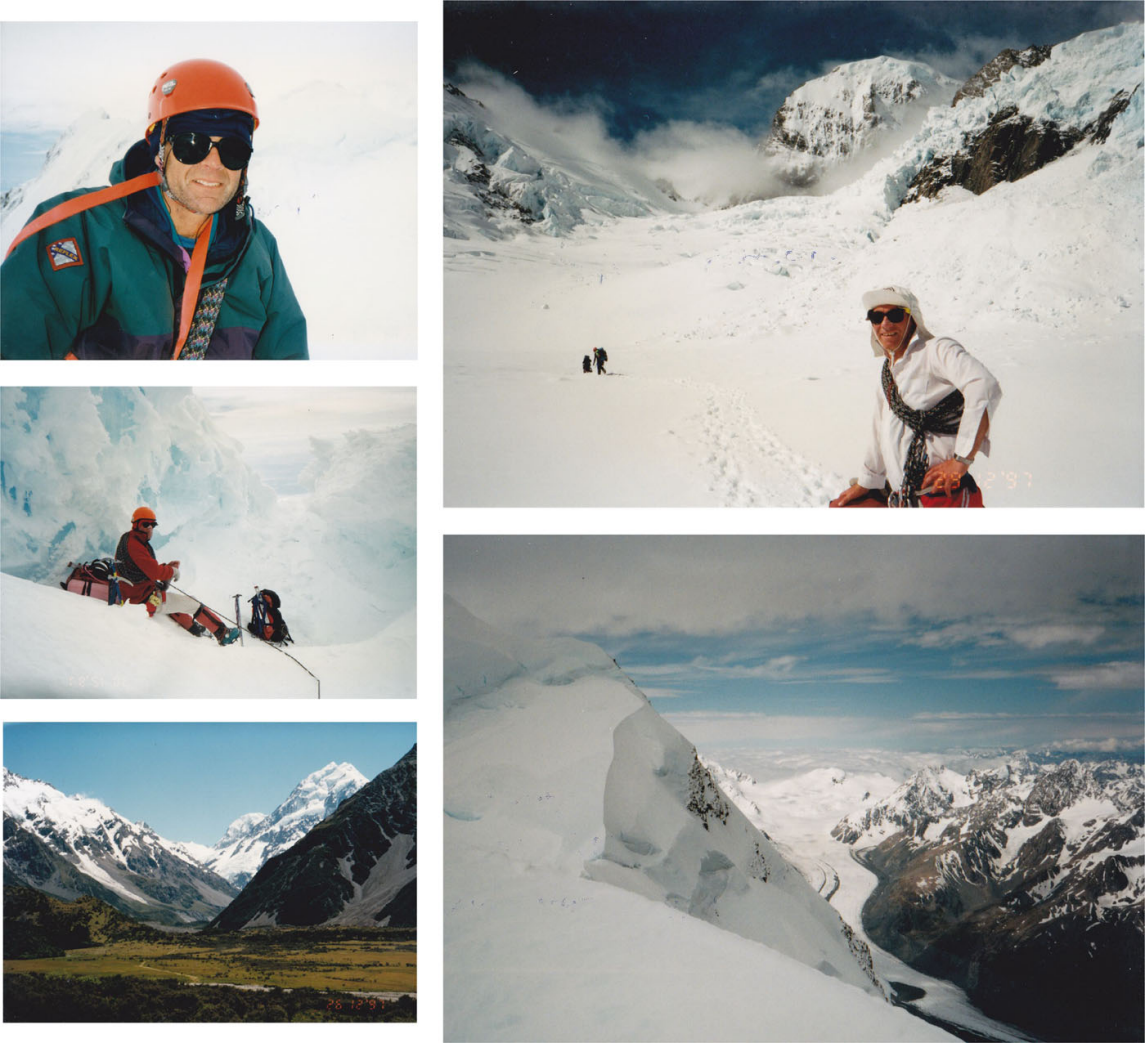
Expedition photos from Mr G, 3 December 1997.

The accident occurred when Mr G and climbing partner were descending from Aoraki Mt Cook Low Peak (30 December 1997; Figure 3). Mt Cook is the highest mountain in New Zealand, standing at 3724 m, with Low Peak at 3593 m. It is considered an extremely difficult mountaineering challenge. On the day of the accident, the forecast was fine. Mr G and partner began their ascent at 3:30 am from Empress Hut, and after summiting they descended via the North West Couloir in the dark (∼9 pm). Towards the bottom of the Couloir, a slab of ice broke away from under them. Both climbers somersaulted down, over a crevasse, and landed on a less steep section. After regrouping, Mr G lost consciousness and when he came to, he could feel some bleeding inside his left trouser leg from his leg wound. His partner packed the wound with his shirt and used a bandage to compress it. The climbers dug and sheltered in a small snow cave until daylight, upon which Mr G's partner returned to the hut to raise the alarm. The climbers were rescued by helicopter and transferred to an ambulance in Mt Cook Village. In the nearby town of Twizel Mr G's wounds were stitched by a local GP, but further repairs (to sinus) were not completed until the New Year. According to the patient, the leg was not examined or followed up again. Mr G gave written consent for the publication of his story and images.

## 3 FINDINGS

**FIGURE 4:**
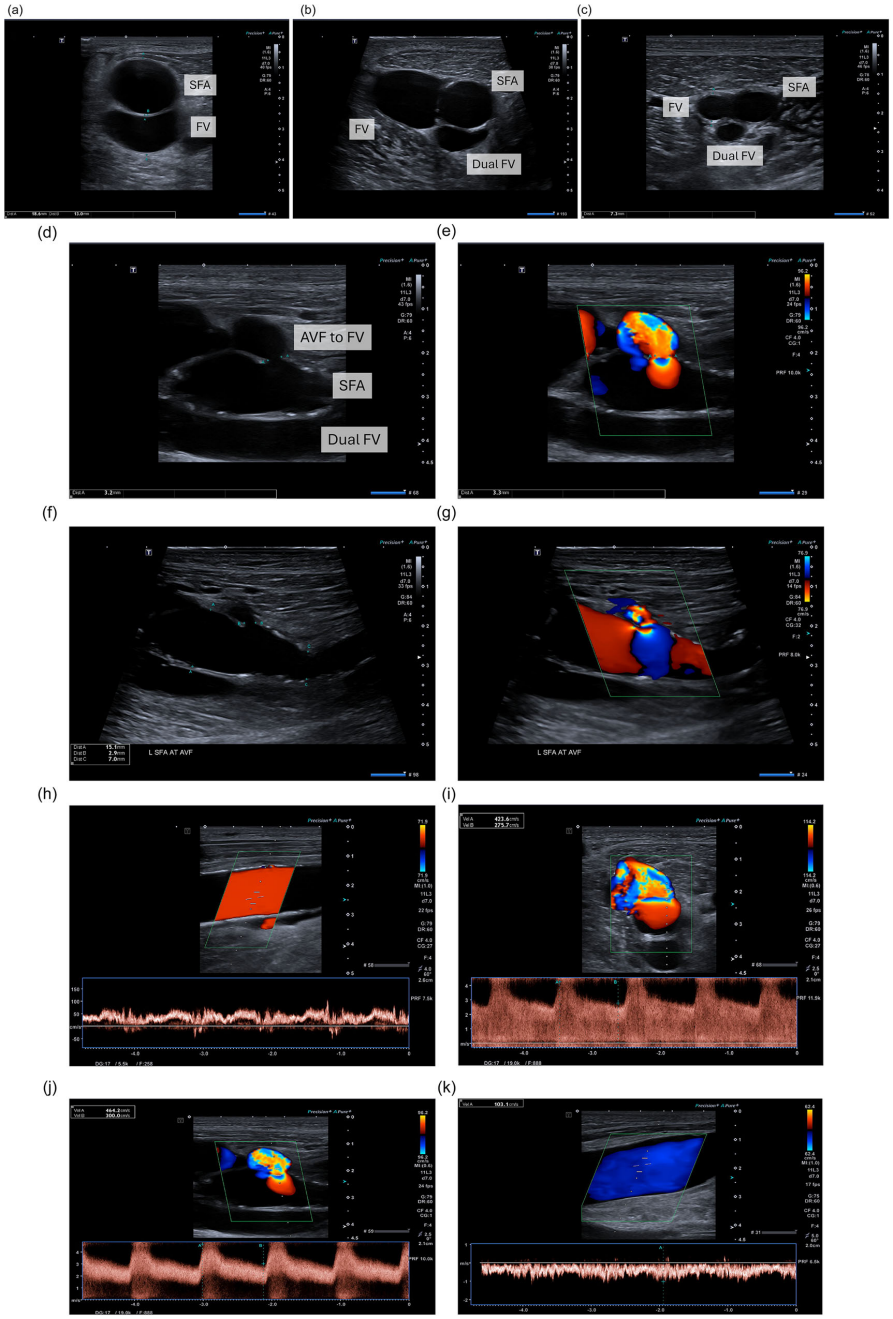
(a–c) Transverse B‐mode ultrasound images demonstrating leg vessel sizes proximal to the arteriovenous fistula (a), at the level of the arteriovenous fistula (b), and (c) the right leg vessel sizes. FV, femoral vein; SFA, superficial femoral artery. (d, e) Longitudinal B‐mode ultrasound image depicting arteriovenous fistula (d) and the fistula in colour (e). AVF, arteriovenous fistula; FV, femoral vein; SFA, superficial femoral artery. (f, g) B‐mode ultrasound image depicting diameters at and around the arteriovenous fistula (f), and the fistula in colour (g). (h–k) Duplex ultrasound image showing the spectral waveform in the superficial femoral artery (h), at the arteriovenous fistula in transverse section (i), and in long section (j), and in the femoral vein (k).

A lower limb ultrasound was performed (11L3, Canon Aplio i700, Canon Medical Systems Corporation, Ōtawara, Japan). Measurements of the diameter and velocity were obtained in the SFA and dual FV bilaterally, along with detailed images of the AVF (Figure 4). The left SFA was markedly dilated (ø 16–19 mm vs. 7 mm on the right); as was the left femoral vein (ø 13 mm vs. 7 mm). In the proximal thigh, an active AVF was observed between the SFA and one of the FVs, with a neck diameter of 2.9–3.3 mm. The SFA tapered significantly from 15 mm over a short length across the site of the AVF to 7 mm distal to the AVF. The velocity waveform in the SFA proximal to the AVF demonstrated disorganised, multidirectional flow consistent with turbulence and a low‐resistance downstream vascular bed (Figure 4h). The flow through the AVF was extremely low resistance, characterised by key features including continuous forward flow throughout the cardiac cycle, high peak systolic and end diastolic velocities (420–462 cm/s and 275–350 cm/s respectively), and a broad systolic peak (Figure 4i‐j). The waveform in the FV showed continuous, turbulent flow through the cardiac cycle (Figure 4k). The FV was large calibre with a duplicated section. All proximal deep veins were also larger calibre than the right side, and significant varicosities were noted to the posterior and medial left thigh.

## 4 TIMELINE

**FIGURE 5:**
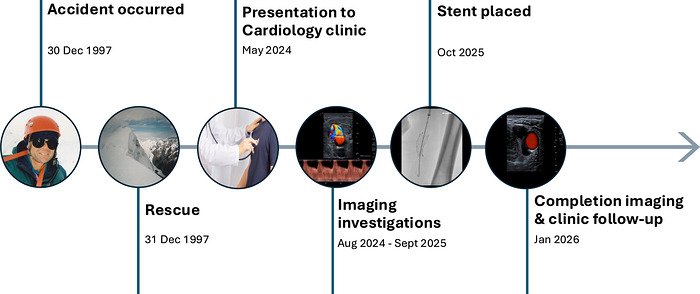
Timeline of care.

The timeline of care is shown in Figure 5.

## 5 DIAGNOSTIC ASSESSMENT

### 5.1 Arterial function testing

On account of the many years of altered haemodynamics in the left leg, we utilised the opportunity to assess the arterial vasodilatory function in both legs for a within‐subject comparison of the chronic effect of such haemodynamics.

#### 5.1.1 Background

Shear stress is the force of the blood flow against the vessel wall, and it provides a key physiological stimulus for vascular adaptation (Deng et al., 2025; Laughlin et al., 2008). Prolonged exposure to high shear (e.g., in surgically created AVFs) leads to significant remodelling of the arterial diameter (Ene‐Iordache et al., 2003; Girerd et al., 1996). Outward remodelling in response to high flow is a homeostatic mechanism to normalise shear rate exposure (Deng et al., 2025). Baseline diameter is inversely correlated with vasodilatory function; partly because flow‐mediated dilation (FMD) is mathematically dependent on baseline diameter (Atkinson & Batterham, 2013), and partly because larger vessels are associated with a lower shear stimulus, resulting in a reduced vasodilatory response (Jazuli & Pyke, 2011). With Mr G, given the AVF on the left side with 27 years of exposure to different flow and shear profiles, we had a rare and unique opportunity to assess the effect of extremely long duration, continuous exposure to higher shear stress in the left versus right SFA. In concordance with a larger left SFA (due to remodelling), our hypothesis was that the vessel would demonstrate a lesser hyperaemic stimulus, and a dampened vasodilatory response.

#### 5.1.2 Methods

Using a linear transducer (15 MHz, Terason uSmart t3300, Teratech, Burlington, MA, USA), FMD was performed on the right and then the left leg, according to published guidelines (Thijssen et al., 2019). Briefly, this entails inflating a cuff around the distal thigh to 200 mmHg (below the AVF on the left; CC17 contoured leg cuff, E20 Rapid Cuff Inflator and AG101 Cuff Inflator Air Source, Hokanson, Bellevue WA, USA). Occlusion was maintained for 5 min. Arterial diameter was recorded for 2 min of baseline, and for 3 min following rapid release of the cuff (<2 s). Time‐averaged velocity, net shear rate and flow were also measured simultaneously (shear rate = 4 × (peak velocity)/diameter; flow = π × (^1^/_2_ diameter/2)^2^ (Deng et al., 2025) × peak velocity/2 × 60). Diameter was determined automatically using edge‐detection software (Cardiovascular Suite v4, Quipu, Pisa, Italy). FMD was calculated as the percentage increase in diameter from baseline.

#### 5.1.3 Results

At rest, the left SFA was approximately 2.5 times larger in diameter than the right, suggesting extensive remodelling, and consequently baseline shear rate was more than double that in the right SFA (Table 1). The flow through the left SFA was considerably higher than the right, as would be expected with a large AVF shunting flow into the low‐resistance venous system.

**TABLE 1:** Comparative diameters and baseline shear rates of left and right superficial femoral arteries at rest.

	Right	Left
**Baseline**		
Diameter (mm)	6.9	16.9
Velocity (cm/s)	10	59
Net shear rate (/s)	61	140
Flow (mL/min)	117	3988
**FMD**		
Peak diameter (mm)	7.3	16.9
Absolute FMD (mm)	0.4	0.0
Relative FMD (%)	6.2	0.0
SRAUC	10542	1710

Abbreviations: FMD, flow‐mediated dilation; SRAUC, shear rate area under the curve.

**FIGURE 6:**
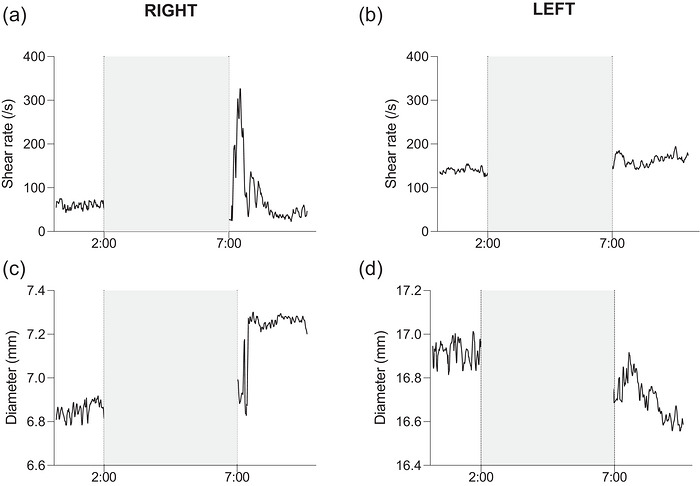
Shear rate (a, b) and diameter responses (c, d) to flow‐mediated dilation in the right and left superficial femoral artery.

The right SFA demonstrated FMD within the normal range (Daniele et al., 2024; Thijssen et al., 2006), whereas the left SFA showed no dilation. Importantly, there was only a minimal increase in shear rate in the left SFA in response to cuff release, indicated by the shear rate area under the curve, which likely contributed to the lack of response, as hyperaemia‐induced increased shear is the stimulus for acute dilation (Figure 6).

## 6 OUTCOMES

### 6.1 Intervention

**FIGURE 7:**
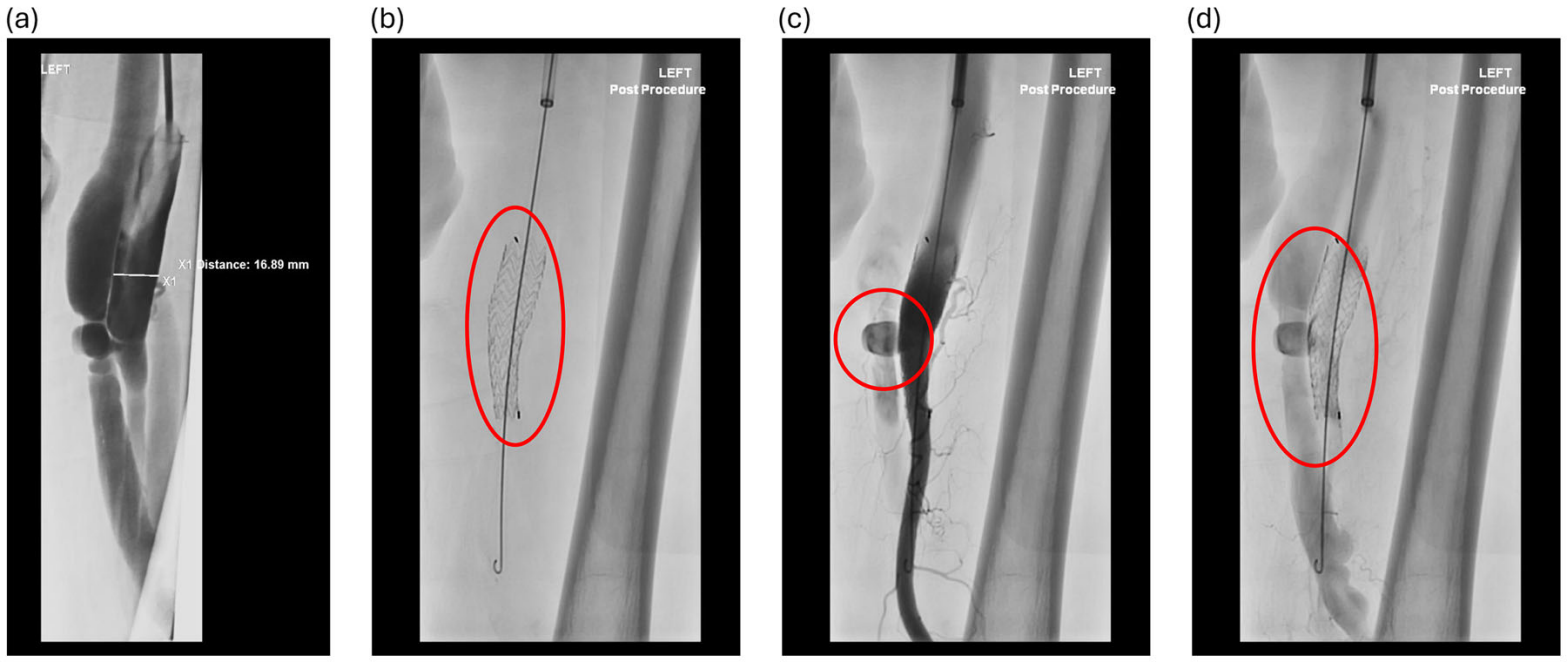
Angiogram images before (a) and during intervention showing stent deployment (b) and residual shunting (c, d).

After discussion at the Vascular and Radiology multi‐disciplinary meeting, Mr G went forward for an endovascular procedure to treat the AVF. A covered stent was placed in the SFA to exclude the AVF (Figure 7). A slightly undersized stent was used as it was the largest available (16 mm tapering to 10 mm, 7 cm long). After stent deployment, a balloon was inflated in the proximal stent to optimise wall apposition. Completion angiogram showed a significant reduction in the amount of arteriovenous shunting with a small amount of residual shunting due to slight stent under‐sizing and incomplete wall apposition.

### 6.2 Follow‐up

**FIGURE 8:**
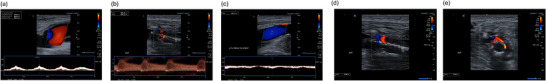
(a–c) Duplex ultrasound images demonstrating the spectral waveform in the superficial femoral artery (a), at the site of residual shunting in the arteriovenous fistula (b) and the femoral vein (c). (d, e) The residual shunting at the arteriovenous fistula in sagittal view (d) and transverse view (e).

**FIGURE 9:**
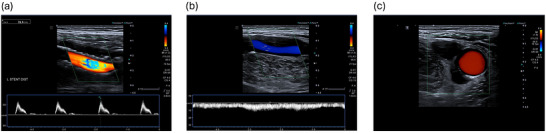
Duplex ultrasound images demonstrating the spectral waveform in the superficial femoral artery stent (a), and the femoral vein (b), with the previous arteriovenous fistula site shown in transverse with no residual shunting (c).

Mr G attended a follow‐up ultrasound appointment 1 week later (Figure 8). The velocity in the SFA remained low resistance and monophasic, but cardiac pulsatility was more evident than in the previous examination. The AVF persisted due to the presence of ongoing shunting; however, the velocity and the size of the fistula both appeared to have reduced (peak systolic velocity 304 cm/s, end‐diastolic velocity 174 cm/s, ø 1.6–2.8 mm). Mr G was again followed up via ultrasound at 3 months’ post‐procedure (Figure 9). The AVF had resolved with no evidence of ongoing shunting. The SFA, including the stented segment, demonstrated normal triphasic flow, and a normal phasic venous waveform was observed in the FV. The patient noted that the left leg swelling had reduced and the thrill was no longer palpable. Mr G was reviewed in the cardiology clinic a few months post‐procedure. Mr G reported a significant improvement in his exercise tolerance and was noted to have reverted to sinus rhythm: the first time in 4 years. Echocardiogram was suggestive of a small improvement in chamber size, even at this early stage after the AVF had sealed completely (approximately 1 month from complete closure to echocardiogram). Further imaging will be pursued in due course to assess for ongoing cardiac remodelling.

## 7 DISCUSSION

Traumatic AVFs are uncommon, and to our knowledge this is the first documented case of an AVF resulting from an ice axe injury, and to go undetected for almost three decades. Most traumatic AVFs in the literature are related to gunshot or bomb blast injuries (Yousuf et al., 2013). While the mean time to discovery of a traumatic AVF has been reported to be just over 2 years, there are many reports of longer latency (Nagra et al., 2025). In this case, the untreated AVF was associated with leg swelling, varicosities and high output cardiac remodelling precipitating arrhythmia and symptoms of early cardiac failure due to the large volume shunt from the arterial to the venous system. Congestive heart failure has previously been associated with chronic AVF, due to the greater cardiac overload (Asensio et al., 2022; Cakmak et al., 2003; Robbs et al., 1994). Whilst the stenting was not a complete success initially, the flow through the shunt had reduced and then spontaneously closed completely culminating in a significant improvement in the patient's symptoms.

This case also provided an opportunity to study the role of chronic, elevated flow and shear stress on arterial and venous adaptation in vivo in humans. Significant remodelling and dilation of both the affected artery and veins had occurred: an extreme example of vascular adaptation. However, as a result of the substantial arterial enlargement, the usual stimulus of 5‐min ischaemia did not induce an increase in flow following cuff release (∼30% increase in the left vs. a 5‐fold increase in the right SFA). The FMD test therefore failed to elicit an adequate shear stimulus, so it is not surprising there was effectively no flow‐mediated dilation in the left SFA. A larger stimulus may have been required to induce vasodilation in this larger artery (Ku et al., 2014); however, even matched stimuli have been shown to induce unmatched responses in different sized vessels (Jazuli & Pyke, 2011). Mr G's between‐leg FMD comparison was insufficient to reveal a meaningful difference in artery function despite the obvious structural adaptation.

The femoral vein was also substantially larger than the contralateral side. Venous adaptation is evident in vein grafts (Fillinger et al., 1994), and has been documented to a lesser extent in athletes, manifesting as deep and perforator vein remodelling (Thomas et al., 2023). Higher pressures and flows are proposed to be integral in the mechanism of adaptation in veins too (Kölegård & Eiken, 2011; Kuk et al., 2021; Muto et al., 2010; Pyle et al., 2010).

This case illustrates the clinical complications associated with an untreated traumatic AVF, the peculiarity of this case is the significant amount of time that passed between injury and its discovery. This case also provided a rare in vivo model of sustained high flow exposure, offering insight into the long‐term arterial and venous adaptations that occur in vivo. A likely explanation for the delay in clinical manifestation is that the shunt was initially smaller, and gradually increased in size over time with the chronic vascular remodelling and concurrent increase in vessel diameters. The physiological effects may have been quiescent for the first few decades with the patient essentially asymptomatic and leading a normal active life. Similarly, while the cardiac chamber dilation occurred over many years ‐ evidence of a chronic high output state ‐ it likely was not as significant early on. This natural history is consistent with several other case reports with long latency periods with the effects of high output cardiac state with or without decompensation eventually leading to medical detection.

### 7.1 Limitations

It is difficult to establish the timeline of progression of the AVF with so many years passing between the accident and first presentation. The flow measured during these assessments is not representative of the flow throughout the whole time period, rather this would have increased progressively as the AVF enlarged. The methodological inadequacy of the FMD test was highlighted in this setting, as the test could not elicit a shear stimulus in a markedly remodelled vessel. We conclude that FMD does not capture the relevant features of adaptation in this setting.

## AUTHOR CONTRIBUTIONS

Kate N. Thomas: Conception of work. Kate N. Thomas, Emma M. W. Jones, Arunesh Majumder, Peter W. Abcarian, Peter J. McLeod: Acquisition, analysis, interpretation, drafting and revising manuscript. All authors have read and approved the final version of this manuscript and agree to be accountable for all aspects of the work in ensuring that questions related to the accuracy or integrity of any part of the work are appropriately investigated and resolved. All persons designated as authors qualify for authorship, and all those who qualify for authorship are listed.

## CONFLICT OF INTEREST

The authors declare no conflicts of interest.

## FUNDING INFORMATION

None.

## GENERATIVE AI STATEMENT

No generative AI tools were used in the preparation of this manuscript.

## Data Availability

No datasets were generated or analysed for this study. All relevant information is included in the article.
